# Zn-Loaded and Calcium Phosphate-Coated Degradable Silica Nanoparticles Can Effectively Promote Osteogenesis in Human Mesenchymal Stem Cells

**DOI:** 10.3390/nano12172918

**Published:** 2022-08-24

**Authors:** Pichaporn Sutthavas, Matthias Schumacher, Kai Zheng, Pamela Habibović, Aldo Roberto Boccaccini, Sabine van Rijt

**Affiliations:** 1 Department of Instructive Biomaterials Engineering, MERLN Institute for Technology-Inspired Regenerative Medicine, Maastricht University, P.O. Box 616, 6200 MD Maastricht, The Netherlands; 2 Jiangsu Province Engineering Research Center of Stomatological Translational Medicine, Nanjing Medical University, Nanjing 210029, China; 3 Institute of Biomaterials, University of Erlangen-Nuremberg, 91058 Erlangen, Germany

**Keywords:** silica nanoparticles, bioactive glass, bone regeneration, zinc, inorganic ion doping, ceramics

## Abstract

Nanoparticles such as mesoporous bioactive glasses (MBGs) and mesoporous silica nanoparticles (MSN) are promising for use in bone regeneration applications due to their inherent bioactivity. Doping silica nanoparticles with bioinorganic ions could further enhance their biological performance. For example, zinc (Zn) is often used as an additive because it plays an important role in bone formation and development. Local delivery and dose control are important aspects of its therapeutic application. In this work, we investigated how Zn incorporation in MSN and MBG nanoparticles impacts their ability to promote human mesenchymal stem cell (hMSC) osteogenesis and mineralization in vitro. Zn ions were incorporated in three different ways; within the matrix, on the surface or in the mesopores. The nanoparticles were further coated with a calcium phosphate (CaP) layer to allow pH-responsive delivery of the ions. We demonstrate that the Zn incorporation amount and ion release profile affect the nanoparticle’s ability to stimulate osteogenesis in hMSCs. Specifically, we show that the nanoparticles that contain rapid Zn release profiles and a degradable silica matrix were most effective in inducing hMSC differentiation. Moreover, cells cultured in the presence of nanoparticle-containing media resulted in the highest induction of alkaline phosphate (ALP) activity, followed by culturing hMSC on nanoparticles immobilized on the surface as films. Exposure to nanoparticle-conditioned media did not increase ALP activity in hMSCs. In summary, Zn incorporation mode and nanoparticle application play an important role in determining the bioactivity of ion-doped silica nanoparticles.

## 1. Introduction

Bone defects caused by trauma, tumor removal and infections are a tremendous burden on western healthcare systems. While autografts remain the gold standard for the treatment of bone defects in patients, issues such as limited availability and complications at the harvest site give rise to the development of synthetic alternatives [1]. Biomaterials such as calcium phosphate ceramics and bioactive glasses are considered promising biomaterials for bone regeneration since they are inherently bioactive and cost-efficient to produce [2,3,4]. Nevertheless, the regenerative capabilities of synthetic biomaterials are considered inferior to natural bone. Doping ceramic biomaterials with bioinorganic ions is an attractive approach to further improve their biological performance since ions are cheap, stable and play vital roles in bone regeneration processes [5,6]. One such example is zinc (Zn), which is an essential trace element found in bones where it plays an important role in bone development, formation and maintenance [7,8]. Lack of Zn is associated with devastating diseases such as osteoporosis, dwarfism and inhibited bone development [9]. In vitro studies have shown that the stimulatory effect of Zn is highly dose-dependent. Specifically, Zn ions applied in the range of 2–5 μg mL^−1^ can stimulate hMSCs’ adhesion and proliferation, whereas higher Zn doses of 15 μg mL^−1^ negatively affect osteogenic differentiation and can lead to cell apoptosis [10]. While ion delivery and dose control are important aspects of the therapeutic application of Zn, the same is true for other ions as well. To date, several methods have been explored to allow controlled and localized ion delivery to guide bone regenerative processes while limiting harmful side effects, including the use of nano and microparticles [11,12]. In this regard, mesoporous silica (MSNs) and mesoporous bioactive glass (MBGs) nanoparticles gained interest for use in bone applications as they are inherently bioactive [13]. For example, MBGs are known to promote calcium phosphate mineralization in the presence of high calcium and phosphate-containing buffers. Moreover, their ordered mesoporous structure makes these nanoparticles promising tools for drug delivery purposes in various biomedical fields [14,15].

Bioinorganic ion delivery using MBGs is well established in the field [16], and there are also several examples regarding the use of MSNs for ion delivery. MSNs and MBGs are both synthesized using sol-gel processes where micelles act as structure-directing agents (templates), creating the mesoporous structure. While the MSN network consists solely of Si-O bonds, MBGs contain network modifiers such as CaO, creating an open glass structure [17,18]. Generally, in biological fluids, MSNs are relatively stable towards biodegradation when no other network modifiers are present [19], whereas MBGs are biodegradable in physiological conditions due to their open glass structure. MSNs and MBGs can be modified to carry multiple bioinorganic ions via incorporation within the silica network or loading in the 2–6 nm wide mesopores. Moreover, ions can be deposited on the nanoparticle surface [20,21].

Considering the dependency of Zn dose on its biological efficacy, we set out to investigate how different ion incorporation methods influence MSN and MBG ability to promote hMSC osteogenic differentiation. Recently, we have shown that MSNs coated with a CaP layer can be used to efficiently deliver multiple ions to hMSCs and that multiple ion delivery is beneficial in stimulating in vitro osteogenesis [21]. In these systems, the CaP layer can act as a pH-sensitive coat, triggering fast ion release at acidic pH (~pH 5) such as those found in endocytic vesicles, while remaining stable at neutral pH.

In this work, we hypothesize that the ion incorporation mode within mesoporous nanoparticles and nanoparticle degradability affects their ability to promote osteogenesis in hMSCs in vitro. To investigate this, four nanoparticle groups were created containing a CaP surface layer and incorporating Zn ions either in the mesopores, on the surface or within the matrix. Specifically, Zn was incorporated within the mesopores of the MSNs and coated with a CaP surface layer to create MSN_Zn_-CaP nanoparticles. The second group also contained Zn in the mesopores and in addition, contained an impurity within the silica network resulting in degradable MSNs in neutral aqueous conditions (DMSN_Zn_-CaP). In the third group, the Zn was deposited together with CaP on the surface to create MSN-CaZnP. The fourth group consisted of MBGs containing Zn within the silica network and surface-modified with a CaP layer to create MBGZn-CaP. Using these four nanoparticle constructs, we aimed to investigate how Zn incorporation amount and release from MSN-CaP and MBG-CaP nanoparticles impact their ability to promote ALP activity as a marker for hMSC osteogenesis and mineralization to assess matrix deposition. We tested three nanoparticle administration routes: exposure of cultured hMSCs to nanoparticle-containing media, hMSCs cultured on top of nanoparticle films or hMSCs exposed to nanoparticle-conditioned media.

## 2. Materials and Methods

### 2.1. Materials

Cetyltrimethylammonium chloride (CTAC), triethanolamine (TEA), tetraethyl orthosilicate (TEOS), (3-Mercaptopropyl) trimethoxysilane (MPTES), 3-aminopropyl triethoxysilane (APTES), ammonium fluoride, phosphate-buffered saline (PBS), fetal bovine serum (FBS), ammonium nitrate (NH_4_NO_3_), N,N-dimethylformamide (DMF), ascorbic acid, MTT (3-(4,5-dimethylthiazol-2-yl)-2,5-diphenyl tetrazolium bromide), anhydrous toluene and N,N-bis(carboxymethyl)aminomethyl were purchased from Sigma Aldrich GmbH (Taufkirchen, Germany). Sodium alizarin sulfonate (Alizarin Red S), Triton X-100, paraformaldehyde (PFA), Tween-20, Bovine serum albumin (BSA), L-glutamine, Trypsin and minimum essential medium alpha (αMEM) were purchased from Fisher Scientific (Landsmeer, The Netherlands). Penicillin and streptomycin were purchased from Gibco Life Technologies (Waltham, MA, USA). Absolute ethanol, ICP-MS graded 60 V/V% Nitric acid (HNO_3_) and ICP-MS graded 37 V/V% hydrochloric acid (HCl) were purchased from VWR (Amsterdam, The Netherlands).

### 2.2. Synthesis of MSNs DMSN and MBGZn

Thiol core-functionalized MSNs were synthesized based on a co-condensation method (Figure S1) similar to what was reported previously [22]. Degradable MSNs were synthesized by incorporating a network impurity within the silica network similar to what was reported by Muller and Bein [23]. Zn-doped mesoporous bioactive glass nanoparticles (MBGZn) were synthesized using a sol-gel with a microemulsion-assisted approach [24]. Further details of synthesis and characterization for MSN, DMSN and MBGZn can be found in the Supporting Information (SI).

### 2.3. Synthesis of COOH Modified MSNs and MBGZn (MSN-COOH and MBG-COOH, Respectively)

MSNs and MBGs were the first surfaces modified by post grafting with amine groups; 100 mg of each nanoparticle were added into 200 mL anhydrous toluene, and the mixture was heated to 110 ℃. Under reflux conditions, 1 mL of APTES was introduced under vigorous stirring and left stirring for 24 h. The nanoparticles were collected via centrifugation and washed with absolute ethanol three times. The amine groups were modified to carboxylic acid groups using our previously reported method [25]. In short, 3 g of succinic anhydride were dissolved in 20 mL DMF and left stirring for 30 min at room temperature to allow the complete dissolution of succinic anhydride. In total, 0.2 g of MSNs re-suspended in 30 mL DMF and were added to the succinic anhydride solution and mixed under vigorous stirring at 60 °C for 48 h. The nanoparticles were collected via centrifugation and washed three times with absolute ethanol. The nanoparticles were stored at −20 °C in absolute ethanol for future use.

### 2.4. Zn Ion Loading in Mesopores and Calcium Phosphate Layer Coating w/o Zinc on MSNs and MBGs

To create MSNs containing Zn ions (MSN_Zn_), 100 mg of MSN-COOHs were immersed in 10 mM Zn(NO_3_)_2_ in Milli-Q water for 12 h. The nanoparticles were collected via centrifugation, and the pellets were washed with Milli-Q water. To create calcium phosphate coating, 100 mg of Zn-loaded MSN were re-suspended in 100 mL Milli-Q water at pH 9 (adjusted by NH_4_NO)_3_. Then, 200 µL of 4 µM (NH_4_)_3_PO_4_ were immediately added to the solution and allowed to react for 30 min. Next, 200 µL of a 10:1 molar ratio 6 µM CaNO_3_ to ZnNO_3_ solution were added to the mixture and stirred for another 30 min. Alternating phosphate and calcium ion addition was repeated two more times. The nanoparticles were collected by centrifugation and washed with absolute ethanol three times. Both MSN_Zn_-CaP and MSN-CaZnP were stored in ethanol at −20 °C.

### 2.5. Characterization of the Inorganic Nanoparticles

Nanoparticle size and zeta potential were measured at a concentration of 0.3 µg/mL in ethanol using a Zetasizer Nano (Malvern Panalytical, Malvern, UK). Their morphology and size were further confirmed using transmission electron microscopy (TEM, FEI Tennai G2 Spirit BioTWIN iCorr (G0.201)). To prepare the samples for TEM analysis, 5 µL of 0.3 µg/mL nanoparticles in absolute ethanol were pipetted onto gold TEM grids and left to dry at room temperature for at least 6 h before imaging. The total element composition of the nanoparticles was measured using inductively coupled plasma mass spectrometry (ICP-MS, iCaP Q, Thermo Scientific, Waltham, MA, USA. All inorganic nanoparticles were completely dissolved by aqua regia (nitric acid and hydrochloric acid mixture in a molar ratio of 1:3) overnight. The standard matrix for ICP-MS analysis consisted of aqueous 1% HNO_3_ containing 20 ppb Sc as the internal standard. Digested MSNs and MBGs were diluted 1:10 in a standard matrix and analyzed in normal mode using He as collision gas. The element amount presented in each analysis was based on calculation in comparison to the known concentration curve of standard Si, Zn, Ca and P ions solution in a standard matrix.

### 2.6. Creating and Characterization Nanoparticles Thin Film Formation

Spin-coating was used to create thin nanoparticle-based films. Glass cover slides were first surface-activated with O_2_ plasma treatment at 0.4 bar at 70 W for 1.5 min (Plasma Cleaner, Diener Electronics Femto PCCE). Then, 20 µL of a nanoparticle suspension (50 µg/mL in absolute ethanol) were pipetted onto the central area of the coverslip and immediately spun at 2000 rpm for 20 sec followed by 7000 rpm for 30 sec forming a thin film on the glass slide. Nanoparticle-based films were dried and stored in the dark at room temperature.

### 2.7. Degradation of Nanoparticles in Cacodylate Buffer and Cell Culture Media

MSNs and MBGs degradation in aqueous solutions with pH 5 or pH 7.4 was studied over a 6-day period. In total, 1 mg nanoparticles was incubated in 1 mL cacodylate buffer (4.3 g sodium cacodylate (Na(CH_3_)_2_AsO_2_) in MilliQ water; adjusted for pH with 0.2 M HCl). The degradation of MSNs and MBGs in a basic medium under the cell culture environment (5% CO_2_ in a humidified atmosphere at 37 °C) was also studied after 1, 3, 6, 24, 72 and 144 h. The amount of Si, Zn, Ca and P released from nanoparticles was determined using ICP-MS analysis.

### 2.8. In Vitro Cell Culture

hMSCs (PromoCell) were expanded in basic cell culture media (αMEM supplemented with 0.2 mM ascorbic acid, 2 mM L-glutamine, 100 U/mL penicillin, 100 mg/mL streptomycin and 10% FBS) and the cultures were kept at 5% CO_2_ in a humidified atmosphere at 37 °C. hMSCs at passage 4 were seeded in 12-well cell culture plates at 5000 cells per cm^2^. For experiments on nanoparticle films, hMSCs were seeded at the same seeding density in a non-treated 6-well culture plate. hMSCs were allowed to adhere for 24 h.

Human osteoblast cells (FOB) were purchased from ATCC (hFOB 1.19). Frozen cells were thawed and expanded in specific media consisting of a 1:1 mixture of Ham’s F12 Medium Dulbecco’s Modified Eagle’s Medium and 2.5 mM L-glutamine along with 10% of G418 fetal bovine serum (ATCC), and the cultures were kept at 5% CO_2_ in a humidified atmosphere at 37 °C. Human umbilical vein endothelial cells (HUVEC) purchased from Bio-connect were expanded in endothelial cell growth medium 211–500 (Sigma-Aldrich, St. Louis, MO, USA), and the cultures were kept at 5% CO_2_ in a humidified atmosphere at 37 °C. FOB at passage 4 and HUVEC at passage 6 was seeded in a 96-well plate at 5000 cells per cm^2^. Both FOB and HUVEC were allowed to adhere for 24 h before cytotoxicity experiments.

### 2.9. Cytotoxicity

The cytotoxicity of MSN-CaZnP, MSN_Zn_-CaP, DMSN_Zn_-CaP and MBGZn-CaP in concentrations ranging from 70 to 500 µg/mL was tested using the MTT assay according to the manufacturer’s protocol. In short, at 80–90% cells’ confluency, freshly prepared media with MSNs at different concentrations was added to the cells. Cytotoxicity after 24 and 72 h exposure to MSNs or MBGs was analyzed. At each time point, 10 μL MTT reagent was added to each well and incubated for 4 h. Formed formazan crystals were dissolved by adding 150 μL of acidified isopropanol (Triton-X 100 with isopropanol in the ratio 1:9). Dissolved crystals were quantified immediately by a microplate reader (BIO-RAD microplate reader-550) at an absorbance wavelength of 570 nm. A standard curve of known formazan concentration was used to calculate the average formazan concentration in each condition.

### 2.10. ALP Assay

To evaluate ALP activity in hMSC cells, nanoparticles were added at a concentration of 70 µg/mL in basic medium (no osteogenic stimulants were added). Cell culture medium was refreshed every 3 days. To prepare the conditioned media, nanoparticles were immersed in basic culture medium at 70 µg/mL for 3 days. Centrifugation of the media was then performed to separate nanoparticles from the media, and the conditioned media was added to hMSCs and refreshed every 3 days. The ALP activity was measured after 7, 14 and 21 days for all conditions. hMSCs cultured with osteogenic media were included as a positive control for all samples. Osteogenic differentiation was evaluated by measuring ALP levels on days 7, 14 and 21 of culture. ALP activity was quantified using the CyQuant^®^ Cell Proliferation Assay Kit (Thermo Fisher Scientific, Waltham, MA, USA) and normalized to DNA similar to what we reported previously [21]. Cells were lysed using cell-lysis buffer (provided with the kit, 1:20 in PBS) containing 0.1% (*v/v*) RNAse A (Thermo Fisher Scientific, Waltham, MA, USA), and then freeze–thawing cycles. ALP activity was measured by incubating cell lysates at a 1:5 ratio (volume) with the CDP-star solution (Sigma Aldrich, St. Louis, MO, USA) in a white-bottom 96-well plate for 30 min in the dark at room temperature. Relative absorbance was determined by a microplate reader (BIO-RAD microplate reader-550). Partial cell lysates were used for quantifying total DNA content via GR-dye solution according to the supplier’s instructions. The fluorescent signal was measured with a spectrophotometer at 520 nm. DNA concentration was calculated using a standard curve according to the manufacturer’s protocol. ALP values were normalized to total DNA content per sample. To evaluate the ALP activity of hMSC cells seeded on top of nanoparticle films, a similar protocol was performed with only alteration that the initial volume of RNAse lysis buffer was increased to 1000 µL to ensure full submersion of the films and complete lysis of the cells.

### 2.11. Mineralization

Calcium deposition of hMSCs was assessed using Alizarin Red S (sodium alizarin sulphonate) staining. After 28 days of culture, cells were fixed with 4% PFA. Alizarin Red S was dissolved in bi-distilled water at a concentration of 22 mg/mL, and pH was adjusted to 4.2 with ammonium hydroxide. Prior to staining, cells were rinsed once with PBS, followed by washing twice with bi-distilled water. Alizarin Red S solution was added to the samples and incubated for 15 min at room temperature, followed by washing thrice with bi-distilled water. Quantification was carried out using a destaining method by adding 1 mL of 10% (*w*/*v*) cetylpyridinium chloride (CPC) (Sigma) in 10 mM sodium phosphate (pH 7.0) solution to each well. After 15 min incubation at room temperature, 10 μL from the extracted stain were transferred to a 96-well plate and diluted 10-fold with CPC solution. The violet-colored supernatant was recorded with a microplate reader (CLARIOstar Multimode Microplate Reader, BMG LABTECH, Ortenberg, Germany) at 555 nm.

### 2.12. Statistical Analysis

Statistical analysis was conducted using one- or two-way analysis of variance (2-way ANOVA) followed by Turkey’s multiple comparison. For all figures, error bars indicate one standard deviation with *p*-values: * *p* < 0.033; ** *p* < 0.02; *** *p* < 0.001 unless stated otherwise.

## 3. Results

### 3.1. Synthesis and Characterization of Zn-Incorporated MSNs and MBGs

**Scheme 1:**
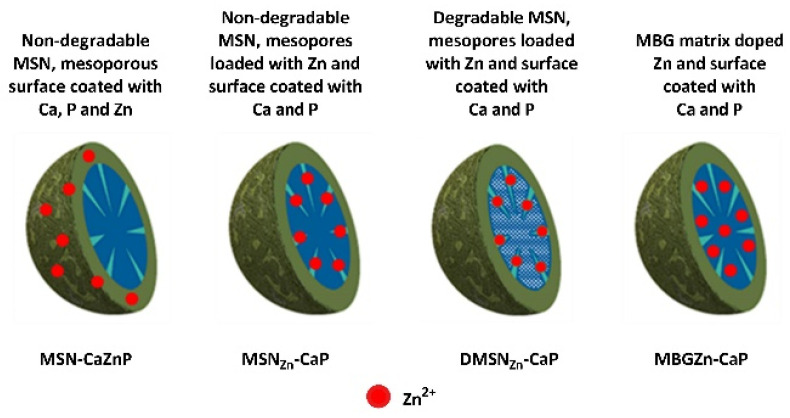
Schematic representation of synthesized nanoparticles and location of Si, Ca, P and Zn ions within the nanoparticles; blue represents Si network, green represents calcium phosphate layer, and red represents Zn ions.

In this study, non-degradable MSNs surface-coated with a CaPZn surface layer, non-degradable MSNs containing Zn ions in the mesopores and a CaP surface layer, biodegradable MSNs containing Zn ions in the mesopores and a CaP surface layer and MBGs containing Zn in the silica matrix and surface coated with a CaP layer were developed (Scheme 1). DMSNs were synthesized by creating an imperfection within the silica network using an adopted co-condensation method, which was first reported by Moller and Bein [23]. Our synthesized DMSN degraded up to 80% of its total mass in an aqueous solution with neutral pH within 6 days (Figure S2). MBGZn synthesis was performed as reported previously [26]. To allow Ca, P and Zn ion deposition on the surface and within mesopores, the MSNs, DMSN and MBGZn were first aminated by postgrafting using APTES. The surface-grafted amines were subsequently modified to carboxylic acid groups by reaction with succinic anhydride, as we reported recently [25]. Zn ions were then incorporated into the mesopores of modified MSNs and DMSNs by physical diffusion to create Zn-loaded MSNs and Zn-loaded DMSNs (MSN_Zn_ and DMSN_Zn_, respectively). The CaP layer was created immediately afterwards to encapsulate the ions within the structure to yield MSN_Zn_-CaP and DMSN_Zn_-CaP by adding Ca and P precursors alternately until a thin layer of CaP was obtained, as we reported previously [21]. In a similar fashion, the Zn-doped CaP-coated surface was created by adding Zn precursors to Ca precursors at a 10% molar ratio during layer formation.

**Figure 1:**
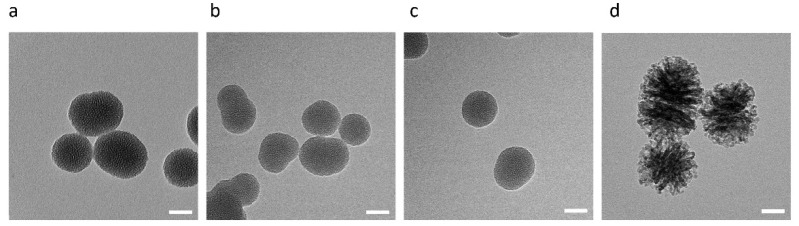
TEM images showing mesoporous structure and nanoparticle size of (**a**) MSN-CaZnP, (**b**) MSN_Zn_-CaP, (**c**) DMSN_Zn_-CaP and (**d**) MBGZn-CaP. Scale bar is 50 nm.

Spherical nanoparticles with ordered mesoporous structures were obtained (Figure 1). CaP modification resulted in a less visible mesoporous structure compared to MSNs and MBGs before CaP coating (Figure S3). Dynamic light scattering (DLS) analysis showed that MSN-CaZnP, MSN_Zn_-CaP and DMSN_Zn_-CaP were similar in hydrodynamic size while MBGZn-CaP were slightly larger than their original size (Table 1 and Table S1). Polydispersity indexes (Pdi) equal to or lesser than 0.3 indicated that all synthesized nanoparticles were monodisperse. All nanoparticles had similar surface potential charges, as shown in Table 1. Crystallinity analysis (XRD) indicated that the calcium phosphate layer on MSN_Zn_-CaP, DMSN_Zn_-CaP and MBGZn-CaP were amorphous and that MSN-CaZnP had a certain level of crystallinity (Figure S4), albeit lower than what is normally observed for crystallized CaP layers [27]. ICP-MS analysis of the digested nanoparticles confirmed the presence of Ca, P, and Zn in all nanoparticles; however, their dose varied (Table 2). In particular, MBGZn had the highest Zn content per mg of nanoparticle, while Zn inside the CaP surface coating was relatively low. Moreover, CaP deposition was much higher on MBG nanoparticles compared to the MSNs, explaining their increased size. In summary, Zn incorporation and CaP layer surface formation were successful, creating four unique nanoparticle constructs.

### 3.2. Ca, P, Zn and Si Release Profiles at Neutral and Acidic pH

**Figure 2:**
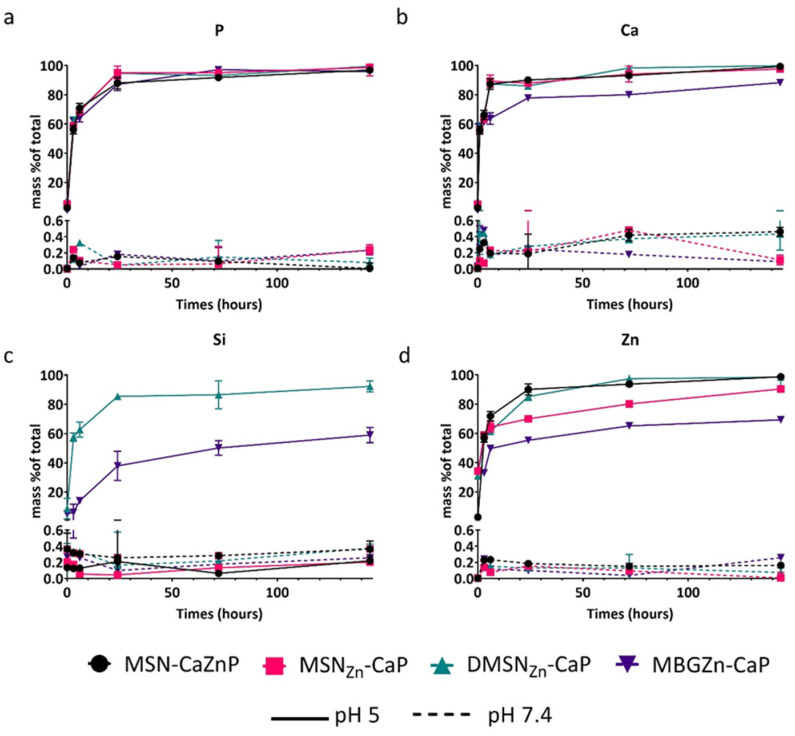
Ion release profiles from MSN-CaZnP (black), MSN_Zn_-CaP (red), DMSN_Zn_-CaP (green) and MBGZn-CaP nanoparticles analysed by ICP-MS; (**a**) P, (**b**) Ca, (**c**) Si and (**d**) Zn ions release in cacodylate buffer pH 5 (line) and pH 7.4 (dashed line).

To determine the stability and ion release capacity of the developed MSNs and MBG nanoparticles, their ion release profile over time was investigated using ICP-MS analysis in a neutral environment (pH 7.4) and acidic environment similar to endosomal vesicles (pH 5). Cacodylate buffer was used for these studies as it does not contain Ca or P. At pH 7.4, Si, Ca, P and Zn ion release was minimal for all four nanoparticles tested (less than 1%, Figure 2 dashed line), showing that the nanoparticles are stable for at least 6 days in buffer at neutral pH. In contrast, at pH 5, rapid dissolution of the CaP coating layer was observed for all nanoparticles. Specifically, total dissolution of phosphate occurred within 24 h of incubation for all four nanoparticles (Figure 2a). The data were fitted to a non-linear best-fit curve to determine the release time required for 50% of the ions to be released (Figure S5, and Tables S2 and S3). Similar rates of calcium ion release could be detected for MSN-CaZnP, MSN_Zn_-CaP and DMSN_Zn_-CaP; the time required for 50% of the ions to be released was in the range of 1.6–1.7 h for all three particles (Figure 2b and Table S3). This was not the case for the MBG nanoparticles, where a rapid release of about 60% of total calcium was observed within the first 4 h, followed by a more sustained release over the 6-day period. This can be explained by rapid calcium release from the CaP surface layer, followed by the slower release of network-incorporated calcium. This is in line with the observed sustained silica matrix dissolution over the 6-day period (Figure 2c). Additionally, DMSN_Zn_-CaP showed dissolution of its silica matrix, which was significantly more rapid compared to MBGZn-CaP particles (3.7 h to release 50% of Si for DMSN_Zn_-CaP compared to 38 h for MBGZn-CaP, Table S3). No silica degradation was observed for the MSN-CaZnP and MSN_Zn_-CaP particles, similar to what we reported recently [21].

Zn ion release profiles at acidic pH were silimar for DMSN_Zn_-CaP, MSN-CaZnP, and MSN_Zn_-CaP and slower for MBGZn-CaP (Figure 2d and Table S3), which may be related to the different modes of Zn incorporation. Specifically, the slower release of Zn in MBG nanoparticles can be explained by its dependency on silica matrix degradation. In contrast, in MSN_Zn_-CaP, the Zn is physically entrapped in the mesopores, which are capped by the CaP composite layer. Dissolution of the CaP is needed to facilitate Zn release in these particles. In addition, Zn ions are absorbed via electrostatic interactions with the negatively charged mesopores, which may further slowdown Zn release. While the Zn was incorporated in a similar manner in DMSN_Zn_-CaP, Zn release from DMSN_Zn_-CaP was significantly faster (Figure 2d). This is likely due to the simultaneous dissolution of the silica network accelerating Zn release. Similar fast release of Zn was observed for MSN-CaZnP, which can be explained by Zn deposition on the surface rather than in the mesopores or silica matrix. Thus, the resulting dissolution rates of the CaZnP layer are similar to CaP layer dissolution.

In summary, here we show that the synthesized nanoparticles are stable at neutral pH and dissolve at acidic pH values. Furthermore, the Zn ion release profiles depend on the mode of ion incorporation. Specifically, CaP and CaZnP surface coatings dissolve rapidly at acidic pH, while matrix or mesoporous incorporated ions are released more slowly.

### 3.3. Stability of the Nanoparticles and Nanoparticles Thin Films in Cell Culture Media

**Figure 3:**
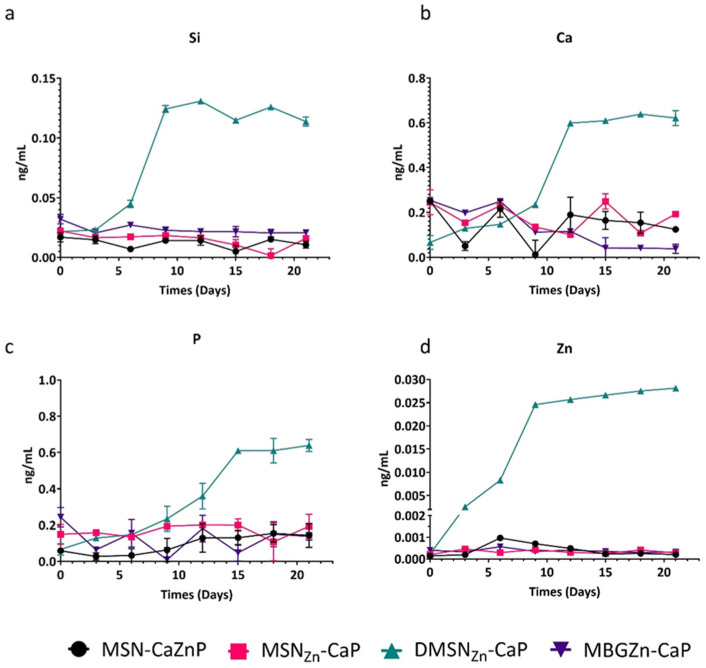
Ion release profiles analyzed using ICP-MS of (**a**) Si, (**b**) Ca, (**c**) P and (**d**) Zn from MSN-CaZnP (black), MSN_Zn_-CaP (red), DMSN_Zn_-CaP (green) and MBGZn-CaP (purple) in cell culture media at 37 °C, 5% CO_2_ in a humidified atmosphere.

To assess nanoparticle stability in cell culture medium, ion release from the nanoparticles was investigated after 21 days. Less than 1% of the total mass of Si, Ca, P and Zn ions were released from MSN-CaZnP, MSN_Zn_-CaP and MBGZn-CaP after the 21-day incubation period. This is in line with our previously obtained results in cacodylate buffer at neutral conditions (Figure 2, dashed line). In contrast, slow ion release that increased over time was observed from DMSN_Zn_-CaP (Figure 3a, green line). Specifically, Si ion release from DMSN_Zn_-CaP became apparent after 6 days of incubation in a cell culture medium and increased until day 9 before levelling off. The release profile of Ca, P and Zn ions followed a similar trend (Figure 3b–d). The dissolution of DMSNs in the cell culture medium was slow; the amount of released Si ions amounted to about 2.58% of the total Si present in DMSNs after 10 days in the cell culture medium. DMSNs contain network impurity making the silica network more prone to hydrolysis.

We also assessed ion release in cell culture medium from the four nanoparticles when deposited as thin films on glass slides. To create these thin films, the nanoparticles were spin-coated on glass slides, as reported recently [21]. In these conditions, no ion release was observed from MSN-CaZnP, MSN_Zn_-CaP and MBGZn-CaP particles over the 21 days (Figure S6). In contrast to our results obtained with DMSN_Zn_-CaP in suspension (Figure 3, green line), DMSN_Zn_-CaP films were also stable as no significant ion release could be observed over the 21-day period (Figure S6, green line).

In summary, MSN-CaZnP, MSN_Zn_-CaP and MBGZn-CaP were stable in cell culture medium over 21 days. DMSN_Zn_-CaP showed slow release of incorporated ions when in suspension in cell culture medium. All nanoparticles were stable in cell culture medium over the 21-day time period when deposited as films.

### 3.4. Nanoparticle Biocompatibility

**Figure 4:**
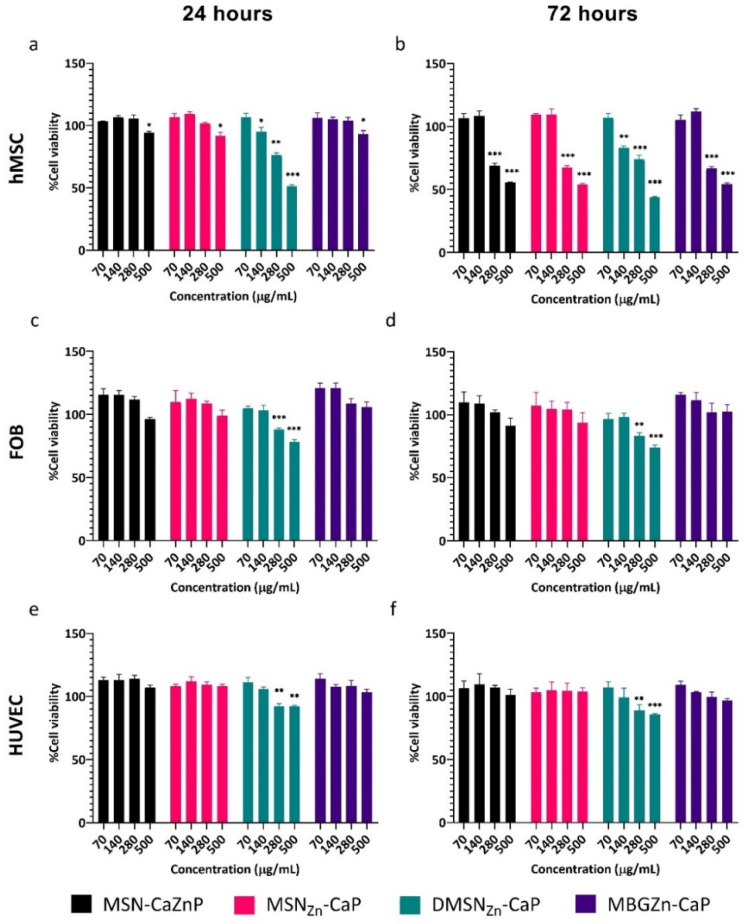
Metabolic activity of hMSC (**a**,**b**), FOB (**c**,**d**) and HUVEC (**e**,**f**) measured using the MTT assay after 24 and 72 h exposure to MSN-CaZnP, MSN_Zn_-CaP, DMSN_Zn_-CaP and MBGZn-CaP to concentrations ranging between 70 and 500 µg/mL. Cell viability is shown as % compared to control cells (not incubated with nanoparticles). Significant differences between samples and control groups are Significant differences between samples and control groups were calculated by two-way ANOVA followed by Turkey’s multiple comparison, * representing *p*-values as follows; * *p* < 0.033; ** *p* < 0.02; *** *p* < 0.001.

Next, the biocompatibility of MSN-CaZnP, MSN_Zn_-CaP, DMSN_Zn_-CaP and MBGZn-CaP to hMSCs at concentrations ranging from 70 to 500 μg/mL was tested in hMSC, FOB and HUVEC cells using the MTT assay. Overall, 24 h exposure to high concentrations (500 μg/mL) of MSN-CaZnP, MSN_Zn_-CaP and MBGZn-CaP resulted in a significant reduction in hMSC cell metabolism compared to control cells (Figure 4a). DMSN_Zn_-CaP nanoparticle concentrations above 140 μg/mL led to a significant decrease in hMSC cell metabolism after 24 h of exposure. Moreover, 72 h of exposure to 280 μg/mL MSN-CaZnP, MSN_Zn_-CaP and MBGZn-CaP led to a significant decrease in hMSCs metabolism, where DMSN_Zn_-CaP significantly affected cell metabolism at 140 μg/mL (Figure 4b).

Exposure to 70–500 µg/mL of MSN-CaZnP, MSNZn-CaP, or MBGZn-CaP after 24 and 72 h did not result in a significant decrease in cell metabolism in either FOB or HUVEC cells (Figure 4c,e). In contrast, 24 h exposure to 280 and 500 µg/mL DMSNZn-CaP led to a significant reduction in FOB cell metabolism with a further decrease after 72 h of exposure compared to control cells. Similar results were found in HUVEC cells, where cell metabolism was significantly decreased after 24 and 72 h exposure to DMSNZn-CaP at concentrations of 280 µg/mL and higher.

In conclusion, hMSCs, FOB and HUVEC viability was not affected by nanoparticle concentrations up to 280 μg/mL when exposed to MSN-CaZnP, MSN_Zn_-CaP or MBGZn-CaP for up to 3 days. However, DMSN_Zn_-CaP concentrations of 140 μg/mL or higher affected hMSCs viability and concentrations of 280 μg/mL and higher affected FOB and HUVEC cells. Based on these findings, 70 μg/mL nanoparticle concentration was selected for further biological studies.

### 3.5. Effect of Zn-Functionalized Nanoparticles on Osteogenic Differentiation

Next, the ability of the nanoparticles to induce osteogenic differentiation by means of measured ALP enzymatic activity was assessed. The osteogenic capabilities of our nanoparticles were tested in basic cell culture conditions (absence of osteogenic stimulators) using three different application methods. In the first application method, the nanoparticles were applied as a suspension in the cell culture medium at a concentration of 70 µg/mL. In the second application method, a nanoparticle-conditioned medium was used in order to assess the effect of ionic degradation products and ion exchange. In the third application method, the nanoparticles were first deposited as thin films on the bottom of cell culture plates, and hMSCs were then cultured on top of these films. hMSCs cultured in osteogenic medium (OM, containing dexamethasone) and in basic medium in the absence of nanoparticles were used as positive and negative controls, respectively.

**Figure 5:**
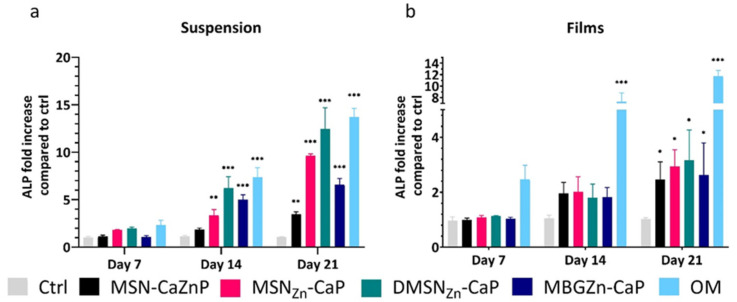
ALP activity in hMSCs normalized to DNA content after exposure to nanoparticles (**a**) to a 70 μg/mL nanoparticle suspension, (**b**) or cultured on thin nanoparticle films. Data are shown relative to controls (cells not exposed to nanoparticles). The experiments were carried out in triplicate. Significant differences between samples and control groups are calculated by one-way ANOVA followed by Turkey’s multiple comparison, * representing *p*-values as follows; * *p* < 0.033; ** *p* < 0.02; *** *p* < 0.001.

Application of the nanoparticles to hMSCs as a suspension in cell culture medium resulted in a significant increase in ALP production after 14 and 21 days for all four nanoparticles. However, the induced ALP levels differed significantly between the tested nanoparticles. Specifically, after 14 days, DMSN_Zn_-CaP and MBGZn-CaP exposure led to significantly higher ALP levels compared to MSN_Zn_-CaP and MSN-CaZnP (Figure 5a). A similar trend was observed after 21 days of exposure, except that DMSN_Zn_-CaP showed similar ALP induction compared to hMSCs cultured in OM and was significantly more effective compared to MBGZn-CaP (*p* = 0.007).

ALP activity of hMSCs cultured on nanoparticle films only increased after 21 days of culture (Figure 5b). Similar ALP levels were observed regardless of the nanoparticle type (2.45-fold for MSN-CaZnP, 2.94-fold for MSN_Zn_-CaP, 3.15-fold for DMSN_Zn_-CaP and 2.64-fold for MBGZn-CaP increase in ALP level compared to negative controls, respectively). The ALP levels were significantly lower compared to those observed after the addition of nanoparticles in suspension. Moreover, hMSCs exposed to conditioned media resulted in no significant increase in ALP activity for any condition (Figure S7).

Overall, hMSCs exposed to nanoparticles in a 70 μg/mL suspension led to high ALP production for all four nanoparticles where DMSN_Zn_-CaP were most efficient. Deposition of the nanoparticles as films only led to significantly increased ALP production after 21 days of exposure, albeit at lower levels compared to suspension conditions.

### 3.6. Mineralization

**Figure 6:**
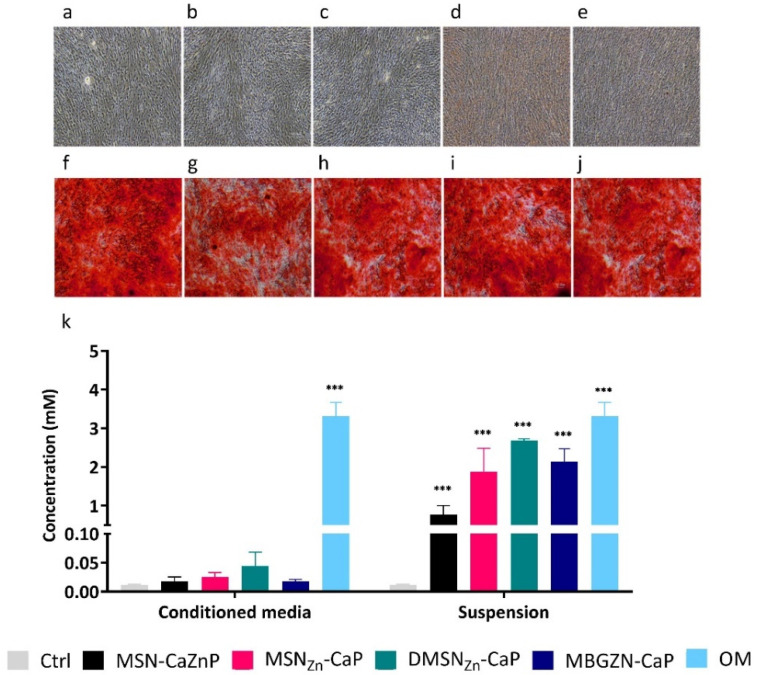
Calcium deposition by hMSCs at day 28 determined by Alizarin Red staining (*n* = 3) when cultured in; (**a**) basic medium (Ctrl); conditioned medium of (**b**) MSN-CaZnP (**c**) MSN_Zn_-CaP (**d**) DMSN_Zn_-CaP and (**e**) MBGZn-CaP; (**f**) osteogenic medium (OM), or in medium supplemented with 70 µg/mL (**g**) MSN-CaZnP, (**h**)MSN_Zn_-CaP, (**i**) DMSN_Zn_-CaP, (**j**) MBGZn-CaP. (**k**) Quantification of calcium content at day 28. Significant differences between samples and control groups are calculated by one-way ANOVA followed by Turkey’s multiple comparison, * representing *p*-values as follows; *** *p* < 0.001.

Next, we investigated whether nanoparticle exposure could promote the ability of hMSCs to mineralize their extracellular matrix. The mineralization of differentiated hMSCs after 28 days in culture was determined using Alizarin Red S Staining. hMSCs cultured in basic and osteogenic media in the absence of nanoparticles were used as negative and positive controls, respectively. Two application methods were considered for these experiments: application of the nanoparticles as suspension and nanoparticle-conditioned media. Limited mineralization could be observed for nanoparticle-conditioned media with DMSN_Zn_-CaP particles (Figure 6d), and no mineralization was observed after exposure to the other nanoparticles (Figure 6b,c,e). In contrast, hMSCs exposed to the nanoparticles in suspension resulted in high mineralization for all conditions compared to the negative control (Figure 6g–j).

## 4. Discussion

In this work, we developed four unique mesoporous ceramic nanoparticles based on MSNs and MBGs containing Zn ions and with a CaP-coated surface. Zn ions were incorporated in three locations in the nanoparticles: in the CaP layer, inside the mesopores or inside the silica matrix. The CaP layer is known to dissolve quickly in acidic conditions such as those found in endosomal vesicles (pH~5) [28] and has been used as a pH-sensitive gating system for the controlled release of therapeutic drugs [29]. In our nanoparticles, CaP surface-coating prevented nanoparticle dissolution in neutral buffer conditions as well as in a cell culture medium. In contrast, MBG nanoparticles without a CaP layer but with similar Zn composition commonly dissolve in neutral aqueous conditions within one week of incubation [26]. Moreover, we showed that DMSN without CaP layers also dissolved in neutral conditions within 6 days, similar to what was observed by others [23]. Thus, surface grafting of CaP layers onto DMSN and MBG nanoparticles can confer stability towards dissolution in aqueous neutral conditions. Conversely, rapid ion release was observed in acidic conditions for all four nanoparticles.

Among the nanoparticles tested, DMSN_Zn_-CaP applied at 140 μg/mL led to reduced hMSCs viability after 72 h incubation. The other nanoparticles only showed a decrease in cell viability upon 72 h incubation when applied at concentrations of 280 μg/mL and higher. This may be due to the relatively high amount of Si ions released from DMSNs in contrast to MSN-CaZnP, MSN_Zn_-CaP or MBGZn-CaP, which contain a more stable silica network. Previously it has been reported that silicate exposure after 24 h incubation at 50 µg/mL or higher is cytotoxic for osteoblast cells [30].

In this study, two methods (ALP activity assay and Alizarin Red S Staining) were used to determine the ability of the developed MSNs and MBG to stimulate osteogenic differentiation in hMSCs. Increased ALP activity in hMSCs is a known marker for osteogenic differentiation due to its role in supporting the mineralization of the cellular matrix during osteogenesis. Moreover, ALP is also involved in the hydrolysis of phosphate esters which provide the phosphate groups required for hydroxyapatite formation [31]. When the nanoparticles were applied as particle suspensions in cell culture medium, high ALP levels in hMSCs were observed. After 14 days, DMSN_Zn_-CaP induced similar ALP activity as our positive control (media supplemented with dexamethasone), followed by MBGZn-CaP nanoparticles. The higher effectiveness of DMSN_Zn_-CaP may be due to the co-delivery of Si ions. Although Si release was also observed from MBGZn-CaP particles, in these nanoparticles, the silica release was much slower compared to DMSN_Zn_-CaP particles. Indeed, both Si and Zn are known to support the growth of bone cells in a dose-dependent manner. Moreover, both soluble Si and SiO_2_ nanoparticles have been shown to induce osteogenic activity in hMSCs at low concentrations. For example, one study reported that solid SiO_2_ nanoparticles could increase ALP activity levels in hMSCs at a concentration of 10 µg/mL [32]. Although MSN_Zn_-CaP did not have a degradable Si matrix, this nanoparticle also led to significantly increased ALP production in hMSCs, whereas MSN-CaZnP were effective to a much lower extent. This difference can be explained by its low Zn content compared to the other three nanoparticles.

After 21 days of incubation, DMSN_Zn_-CaP exposure led to the highest ALP activity, followed by MSN_Zn_-CaP and MBGZn-CaP nanoparticles. The improved effectiveness of MSN_Zn_-CaP over MBGZn-CaP after longer incubation periods may be related to the faster Ca and Zn release profiles for MSN_Zn_-CaP particles compared to MBGZn-CaP. These data indicate that Zn incorporation mode and release profile are important factors in determining the biological activity and ability to promote osteogenesis of Zn-doped ceramic nanoparticles. All four nanoparticles could also significantly promote mineralization where MSN_Zn_-CaP, DMSN_Zn_-CaP and MBGZn-CaP were most and equally effective. The lower effectiveness of MSN-CaZnP in promoting both ALP activity and mineralization is likely related to its lower Zn ion content compared to the other three nanoparticles.

No increase in ALP activity nor mineralization was observed in hMSCs exposed to nanoparticle-conditioned media. This is in line with the observation that all four nanoparticles were stable in cell culture media at neutral conditions, shielded from ion dissolution by the CaP layer. Previously, we showed that MSN-CaP nanoparticles are efficiently internalized by hMSCs, promoting high ion uptake [21]. Taken together, our data indicate MSN-CaP and MBG-CaP cell internalization via endocytosis followed by pH-responsive intracellular ion release as a likely mechanism leading to the induction of hMSC osteogenesis.

hMSCs cultured on stable films prepared from the four nanoparticles also resulted in increased ALP activity, albeit significantly lower compared to nanoparticle suspension conditions, and only after 21 days of culture. Moreover, no difference in ALP induction was observed between the nanoparticles. Considering that the films were stable and no ion release was observed in cell culture media in neutral conditions, this may be explained by the similarity between surface crystallinity and nanoscale surface topography. While the exact mechanism of how CaP surfaces influence the osteogenesis process remains unclear, it has been suggested that the crystallinity, chemical composition and nanoscale topography of CaP surfaces are important factors in inducing osteogenesis in bone cells.

## 5. Conclusions

In this study, we developed four nanoparticles incorporating Zn in the CaP layer, inside the mesopores or inside the silica matrix of MSN or MBG particles. The particles were further surface coated with CaP layers as a pH-sensitive gating system. We demonstrated that CaP surface coating could be used to infer stability to degradable MSNs and MBGs under neutral conditions and stimulate ion release under acidic conditions. The Zn incorporating MSNs and MBGs enabled significant induction of ALP activity and mineralization of hMSCs in the absence of other stimulators. Degradable MSNs with Zn incorporated in the mesopores were equally effective as osteogenic medium, followed by MBGs containing Zn in the silica matrix. The difference in bioactivity between the four nanoparticles is likely related to the Zn incorporation amount and release profile in association with Si ion release. Comparison between the modes of nanoparticle application revealed that Zn incorporated silica nanoparticles were more effective in inducing ALP activity when applied as a suspension compared to stable thin films of the same materials or nanoparticle-conditioned media. Moreover, our data show that Zn ion incorporation mode and release rate are important factors in the bioactivity of MSNs and MBGs when applied as nanoparticle suspensions and should be considered in the design of ion-doped ceramic nanoparticles to improve their therapeutic use.

## Figures and Tables

**Table 1:** Hydrodynamic size, Pdi and surface zeta potential of MSN-CaZnP, MSN_Zn_-CaP, DMSN_Zn_-CaP and MBGZn-CaP measured by dynamic light scatting. All nanoparticles were measured in distilled water.

Sample	Size (nm)	Pdi	Zeta Potential [mV]
MSN-CaZnP	195 ± 15	0.219 ± 0.09	−28 ± 7
MSN_Zn_-CaP	215 ± 9	0.259 ± 0.10	−22 ± 9
DMSN_Zn_-CaP	225 ± 23	0.251 ± 0.13	−26 ± 4
MBGZn-CaP	270 ± 12	0.31 ± 0.11	−19 ± 7

**Table 2:** Ion composition of 100 mg/L of MSN-CaZnP, MSN_Zn_-CaP, DMSN_Zn_-CaP and MBGZn-CaP using ICP-MS analysis.

Sample	Si (mg/L)	Ca (mg/L)	P (mg/L)	Zn (mg/L)
MSN-CaZnP	25.412	0.217	0.143	0.022
MSN_Zn_-CaP	24.704	0.203	0.138	3.833
DMSN_Zn_-CaP	22.976	0.186	0.120	3.230
MBGZn-CaP	23.111	2.317	1.389	9.655

## Data Availability

Not applicable.

## Supplementary Materials

The following supporting information can be downloaded at: www.mdpi.com/article/10.3390/nano12172918/s1. Figure S1: Synthesis scheme of core-labelled MSN, Figure S2: TEM images showing morphology of DMSN_Zn_-CaP and DMSN, and ion release profiles of Si from DMSN and DMSN_Zn_-CaP, Figure S3: TEM images showing morphology of MSN, DMSN and MBGZn, Figure S4: XRD patterns of; MSN-CaZnP, MSN_Zn_-CaP, DMSN_Zn_-CaP and MBGZn-CaP, Figure S5: non-linear fitting curves applied to ion release profiles of MSN, MSN-CaZnP, MSN_Zn_-CaP, DMSN_Zn_-CaP and MBGZn-CaP Figure S6: Ion release profiles of MSN-CaZnP, MSN_Zn_-CaP, DMSN_Zn_-CaP and MBGZn-CaP, and thin nanoparticle films in cell culture medium, Figure S7: Relative ALP activity in hMSCs after exposure to nanoparticles in the form of conditioned media, Table S1: Hydrodynamic size, Pdi and surface potential of MSN-CaZnP, MSN_Zn_-CaP, DMSN_Zn_-CaP and MBGZn-CaP, Table S2: calculated variables from non-linear logarithmic growth model, Table S3: The time required for 50 % of Si, Ca, P and Zn ions to be released from MSN-CaZnP, MSN_Zn_-CaP, DMSN_Zn_-CaP and MBGZn-CaP.
