# Supplementary material for: Zn-Loaded and Calcium Phosphate-Coated Degradable Silica Nanoparticles Can Effectively Promote Osteogenesis in Human Mesenchymal Stem Cells
Source: Nanomaterials (Basel). 2022 Aug 24;12(17):2918. doi: 10.3390/nano12172918 (PMC9457856; doi:10.3390/nano12172918)
Supplement: Supplementary file 1 [file nanomaterials-12-02918-s001.zip › nanomaterials-1822256-supplementary.pdf]

Supplementary data

Zn loaded and Calcium Phosphate-coated Degradable Silica Nanoparticles Can Effectively Promote Osteogenesis in Human Mesenchymal Stem Cells.

Pichaporn Sutthavas[1], Matthias Schumacher[1],Kai Zheng[2], Pamela Habibovic[1], Aldo Boccaccini[3], and Sabine van Rijt[1]*

1 Department of Instructive Biomaterials Engineering, MERLN Institute for Technology-Inspired Regenerative Medicine, Maastricht University, P.O. Box 616, 6200 MD Maastricht, The Netherlands; p.sutthavas@maastrichtuniversity.nl (P.S.); m.schumacher@maastrichtuniversity.nl (M.S.); p.habibovic@maastrichtuniversity.nl (P.H.)
2 Jiangsu Province Engineering Research Center of Stomatological Translational Medicine, Nanjing Medical University, Nanjing 210029, China; kaizheng@njmu.edu.cn (K.Z)
3 Institute of Biomaterials, University of Erlangen-Nuremberg, 91058 Erlangen, Germany; aldo.boccaccini@fau.de (A.B.)
***** Correspondence: s.vanrijt@maastrichtuniversity.nl (S.R.)

* Corresponding author:
Sabine van Rijt, PhD
Assistant Professor
Department of Instructive Biomaterials Engineering
MERLN Institute for Technology-Inspired Regenerative Medicine, Maastricht University
E-mail: s.vanrijt@maastrichtuniversity.nl

S.1 Synthesis of core-labelled MSN

To synthesize MSNs, a mixture of 1.63 g TEOS (7.82 mmol), MPTES (112 mg, 0.48 mmol) and 14.3 g TEA (95.6 mmol) was heated to 90 °C under static conditions (Solution 1). Solution 2 included 100 mg ammonium fluoride (2.70 mmol) dissolved in a solution of 2.41 ml CTAC (1.83 mmol, 25% (wt) in H_2O) and 21.7 ml bi-distilled water (1.21 mmol) by heating to 60 °C. Solution 2 was rapidly added to solution 1, and the mixture was stirred vigorously at 700 rpm for 20 min while left to cool. Then, 138.2 mg TEOS (0.922 mmol) was added in four equal increments (34.55 mg each) every 3 minutes. The solution was then left stirred overnight at room temperature. The particles were then collected by centrifugation at 7800 rpm for 20 min and washed once with ethanol. Template extraction was performed by dispersion into an ammonium nitrate in ethanol solution (2 g NH_4NO_3 in 100 ml ethanol) and refluxed for 45 minutes at 90 °C. MSNs were collected by centrifugation and washed with ethanol before further template extraction in 100 ml of a 3.7% hydrochloric acid solution in ethanol for 45 minutes at 90 °C. MSNs were collected by centrifugation, washed twice with ethanol and

stored in suspension at -20 °C. The thiol groups functionalized core of the MSNs were labelled with ATTO-647-Maleimide to create fluorescent core labeled MSNs.

S.2 Synthesis of MBGZn

Zn-containing mesoporous bioactive glass nanoparticles (MBGZn) were synthesized by using a microemulsion-assisted sol-gel approach[24]. Briefly, 2.24 g hexadecyltrimethylammonium bromide (CTAB) was dissolved in 104 mL of deionized water under continuous stirring at 30 °C for 30 min. Then, 32 mL of ethyl acetate was slowly poured into the solution under continuous stirring for 30 min. Subsequently, ammonium hydroxide (28%) was used to maintain pH at 10.5. After 15 min 23.04 mL of tetraethyl orthosilicate (TEOS) was added to the mixture and stirred for 30 min. Afterwards 4.34 g of $Ca(NO_3)2.4H2O$ was dissolved into the mixture. After 30 min, 1.09 g $Zn(NO_3)2.6H2O$ was introduced into the solution. The nominal composition of Zn-MBGNs was 70SiO2–25CaO–5ZnO (mol%). The mixture was stirred further for 4 h and then the suspension was centrifuged at a rate 9000 rpm (Centrifuge 5430R, Eppendorf, Germany) for 10 min to separate particles from the mixture. The precipitate was then washed twice with water and once with ethanol. Subsequently, the precipitate was dried in an oven at 60 °C for 24 h, followed by calcination at 700 °C for 2 h at a heating rate of 2 °C min−1.All the used chemicals were purchased from Sigma-Aldrich (Darmstadt, Germany) without further purification.

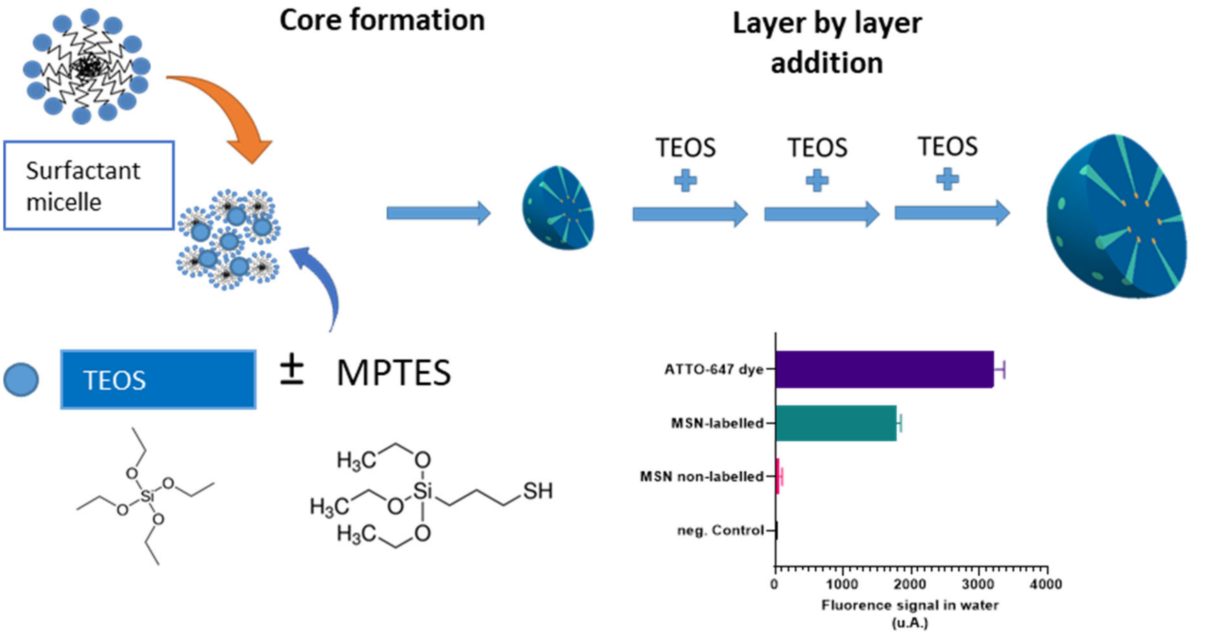

Figure S1: Synthesis scheme of core-labelled MSN. Thiol groups core functionalization MSNs were further confirmed by labelled with ATTO-647-Maleimide and measured in a microplate reader (BIO-RAD) at 647 nm.

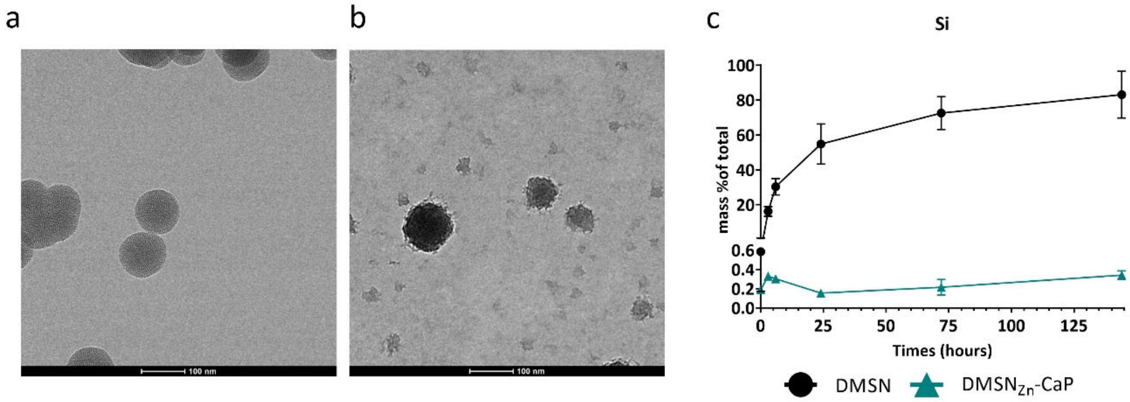

Figure S2: TEM images showing morphology of a) $DMSN_{Zn}$-CaP and b) DMSN after incubation in milliQ water of pH 7.4 for 144 hours. c) Ion release profiles of Si from DMSN and $DMSN_{Zn}$-CaP

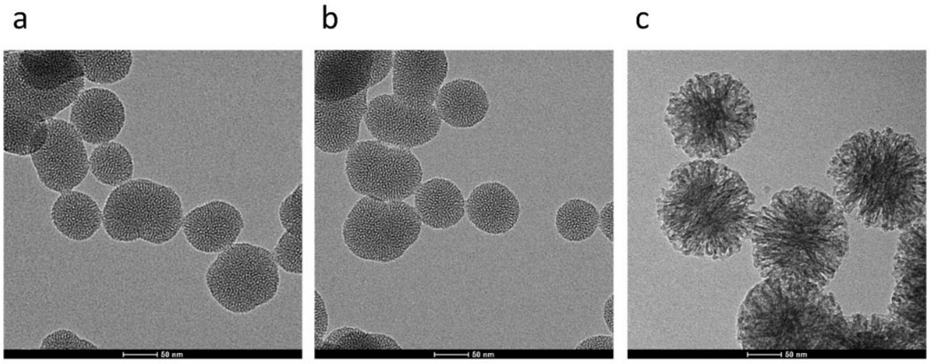

Figure S3: TEM images showing morphology of a) MSN, b) DMSN and c) MBGZn Scale bar is 50 nm.

Table S1 : Hydrodynamic size, Pdi and surface potential of MSN-CaZnP, MSNZn-CaP, DMSNZn-CaP and MBGZn-CaP measured by dynamic light scatting. All nanoparticles were measured in absolute ethanol.

Sample	Size (nm)	Pdi	Zeta potential [mV]
MSN-CaZnP	190±15	0.214±0.03	-19±5
MSN_{Zn}-CaP	198±9	0.247±0.05	-21±3
$DMSN_{Zn}$-CaP	201±22	0.250±0.10	-22±6
MBGZn-CaP	247±12	0.292±0.04	-18±10

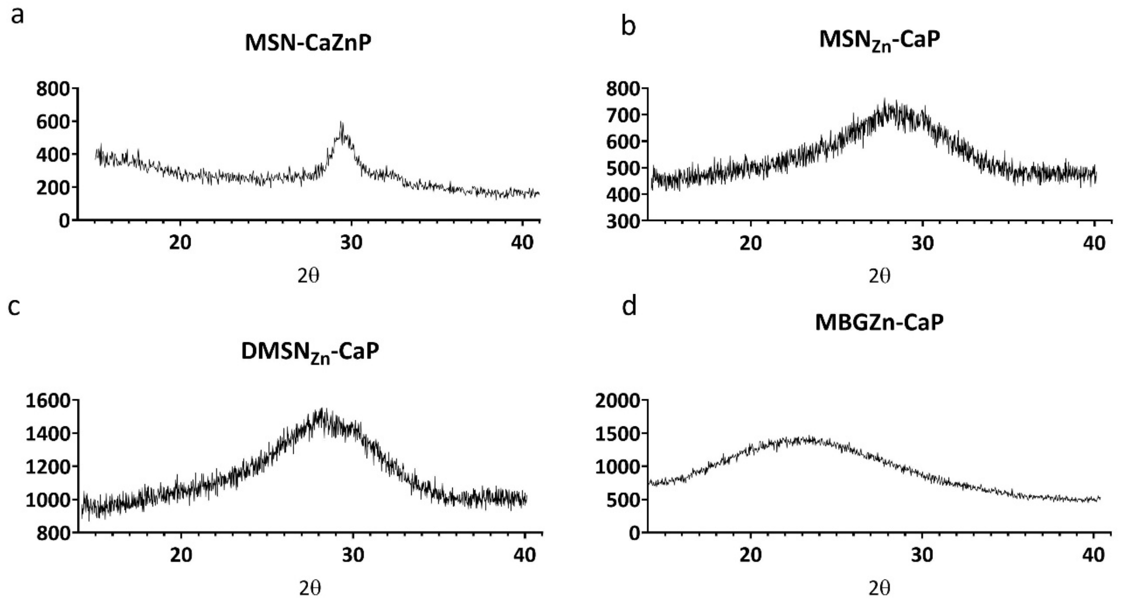

Figure S4 : XRD patterns of displayed amorphous non- crystallization of ; a) MSN-CaZnP, b) MSNZn-CaP, c) DMSNZn-CaP and d) MBGZn-CaP displayed amorphous non- crystallization of all nanoparticles.

S.3 Half-release time calculation

The half-release time required for silica, calcium, phosphate and zinc ions was calculated by fitting the data as displayed in Figure 2 to a non-linear fitting curve. The mathematical equation is shown below. Individual variables for each data sets can be found in Table R2.

$$M\% = YM * \frac{Y0}{(YM-Y0)*exp(-k*t)+Y0} \qquad \textit{Equation 1}$$

Where M% is % of total amount of ions present and t is time (hours).

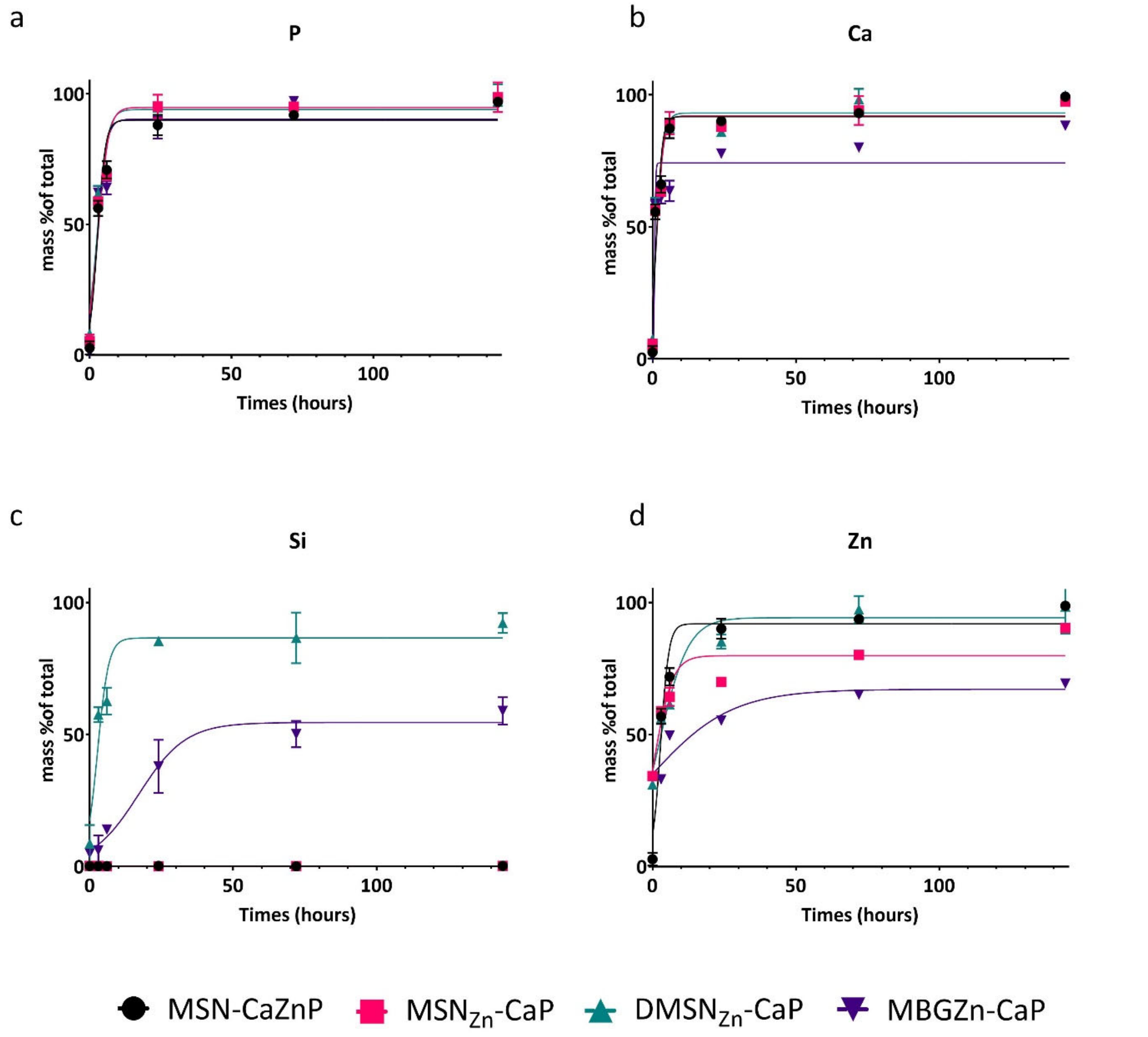

Figure S5: non-linear fitting curves based on equation 1 applied to a) P, b) Ca, c) Si, and d) Zn ion released from MSN-CaZnP, MSNZn-CaP, DMSN$_{Zn}$-CaP and MBGZn-CaP.

Table S2: calculated variables from non-linear logarithmic growth model

Si		MSN-CaZnP	MSN$_{Zn}$-CaP	DMSN$_{Zn}$-CaP	MBG$_{Zn}$-CaP
	YM	N.A.	N.A.	86.51	54.52
	Y0	N.A.	N.A.	17.23	6.052
	k	N.A.	N.A.	0.514	0.122
Ca					
	YM	91.77	92	93.13	74.2
	Y0	19.83	21.67	23.99	1.025
	k	0.9356	0.8144	0.7455	5.551
P					
	YM	89.88	94.79	94.01	90.27
	Y0	11	15.76	17.2	13.57
	k	0.7171	0.5355	0.5528	0.6361

Zn					
	YM	91.92	79.87	94.24	67.12
	Y0	11.64	36.23	35.76	34.95
	k	0.6877	0.318	0.2009	0.07062

The time required for 50% of each ion to be release from the nanoparticles was calculated by solving t in Equation 1 and can be found in Table R3

Table S3: The time required for 50 % of Si , Ca, P and Zn ions to be released from MSN-CaZnP, MSN_{Zn}-CaP, $DMSN_{Zn}$-CaP and MBGZn-CaP. N.A. = non applicable with equation 1

	MSN-CaZnP	MSN_{Zn}-CaP	$DMSN_{Zn}$-CaP	MBG_{Zn}-CaP
Ions	Half release time (hours)			
Si	N.A.	N.A	3.68	38.04
Ca	1.55	1.66	1.60	0.90
P	3.06	3.22	2.93	3.07
Zn	3.08	2.22	3.19	13.90

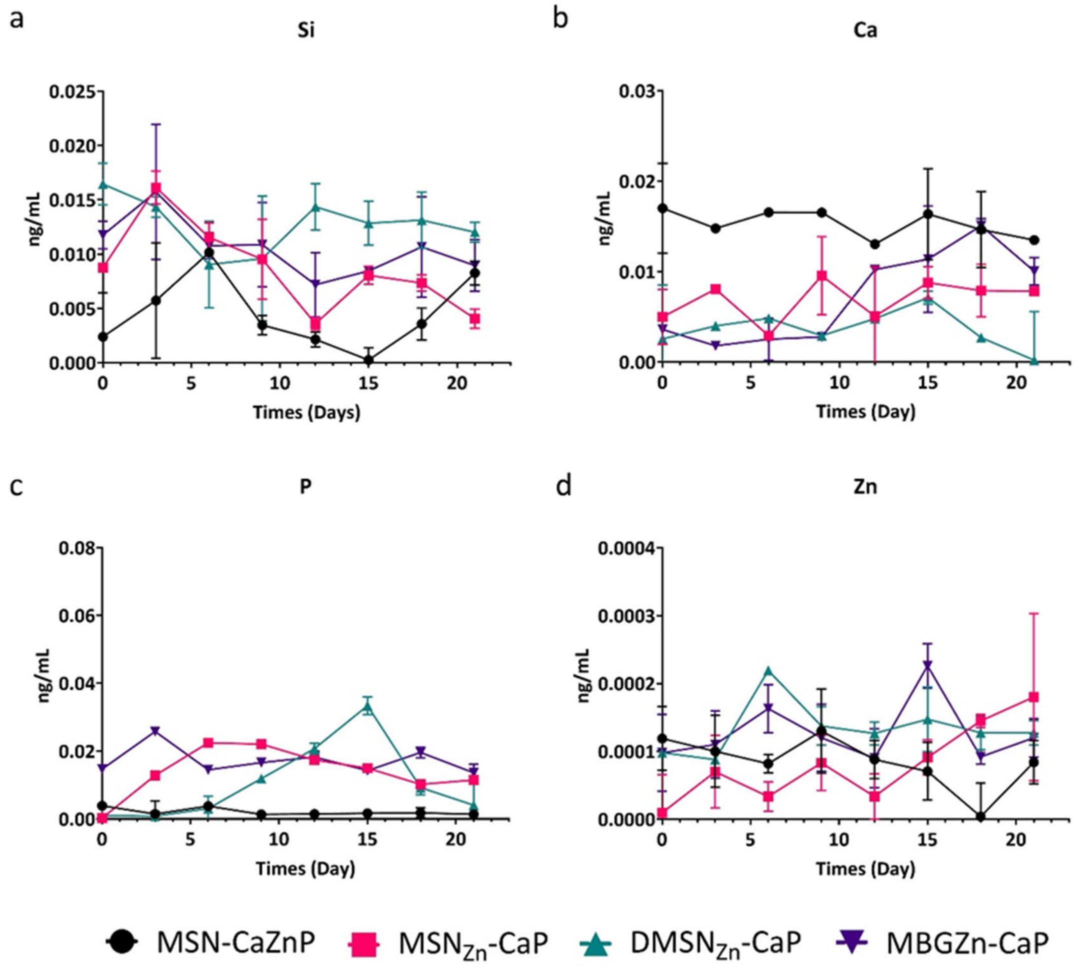

Figure S6: Ion release profiles using ICP-MS of a) Si, b) Ca, c) P and d) Zn of MSN-CaZnP (black), MSN_{Zn}-CaP (red), $DMSN_{Zn}$-CaP (green) and MBGZn-CaP (purple) thin nanoparticles film in cell culture media kept at 37 °C, 5% CO_2 in a humidified atmosphere.

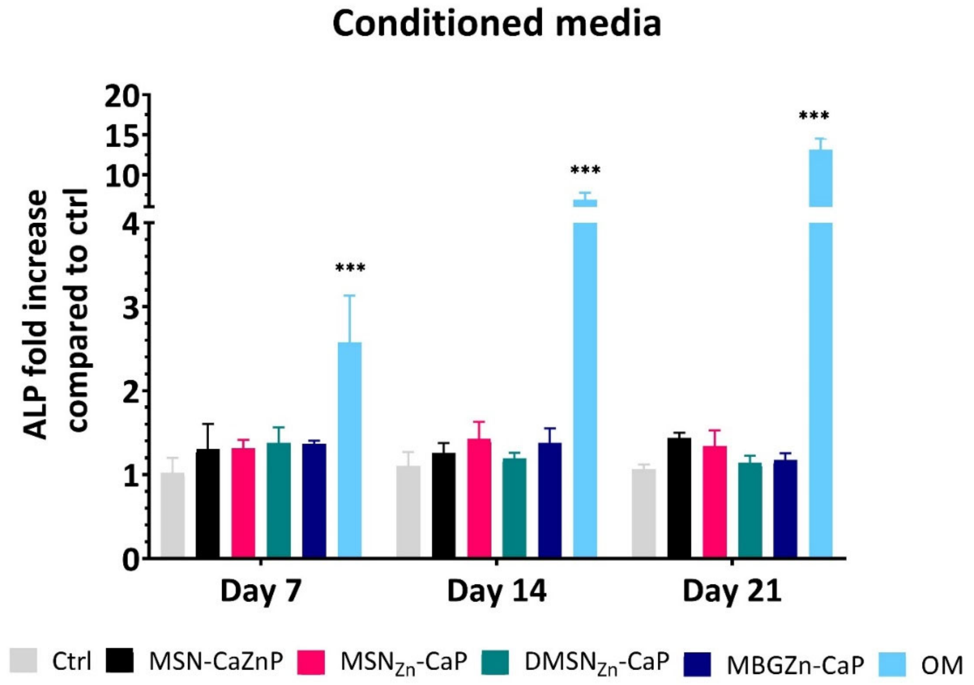

Figure S7: Relative ALP activity in hMSCs after exposure to nanoparticles in the form of conditioned media normalized to DNA content. The experiments were done in triplicate. Significant difference compared to the controls are shown by * representing p-values as follows; *$p < 0.033$; **$p < 0.02$; ***$p < 0.001$.
